# The Physicians' Visiting List

**Published:** 1889-02

**Authors:** 


### Bibliographical.

---

THE PHYSICIANS' VISITING LIST, for 1889, published by Lindsay & Blakiston, Philadelphia, Pa., contains Almanac for 1889 and 1890; Table of Signs to be used in keeping accounts; Marshall Hall's Ready Method in Asphyxia; Poisons and Antidotes; The Metric or French Decimal System of Weights and Measures; Dose Table, revised and rewritten for 1888, by Hobart Amory Hare, M. D., Demonstrator of Therapeutics, University of Pennsylvania; List of New Remedies for 1888, by same author; Aids to Diagnosis and Treatment of Diseases of the Eye, Dr. L. Webster Fox, Clinical Asst., Eye Department Jefferson Medical College, and G. M. Gould; Diagram Showing Eruption of Milk Teeth, Dr. Louis Starr, Professor, of Diseases of Children, University Hospital, Philadelphia; Posological Table, Meadows; Disinfectants and Disinfecting; Examination of Urine, Dr. J. Daland, based upon Tyson's "Practical Examination of Urine," 5th Edition; Incompatibility, Prof. S. O. L. Potter; A New Complete Table for Calculating the Period of Utero-Gestation; Sylvester's Method for Artificial Respiration; Diagram of the Chest; Blank Leaves, suitably ruled, for Visiting List, Monthly Memoranda, Addresses of Patients and others, Addresses of Nurses, their references, etc., Accounts asked for, Memoranda of Wants, Obstetric and Vaccination Engagements, Record of Births and Deaths; Cash Account, etc.

A number of improvements and additions have been made to the reading matter in the first part. This has been done, however, without increasing the number of pages, Great care has been taken in selecting the leather for the covers and in each detail of manufacture.

Perpetual edition, without dates, can be commenced at any time, and used until full. Similar in style, contents and arrangements to the regular edition. No. 1. Containing space for over 1,300 names, with blank page opposite each Visiting List page. Bound in Red Leather cover, with Pocket and Pencil, $1.25. No. 2. Containing space for 2,600 names, with blank page opposite each Visiting List page. Bound like No. 1, with Pocket and Pencil, $1.50.

These lists, without dates, are particularly useful to young physicians unable to estimate the number of patients they may have during the first years of practice, and to physicians in localities where epidemics occur frequently.

